# Transient neuromotor phenotype in transgenic spastic mice expressing low levels of glycine receptor β-subunit: an animal model of startle disease

**DOI:** 10.1046/j.1460-9568.2000.00877.x

**Published:** 2000-01

**Authors:** Lore Becker, Bettina Hartenstein, Johannes Schenkel, Jochen Kuhse, Heinrich Betz, Hans Weiher

**Affiliations:** Institut für DiabetesforschungKölner Platz 1, 80804 München, Germany; 1 Forschungszentrum Karlsruhe, Institut für GenetikPostfach 3640, 76021 Karlsruhe, Germany; 2 Abteilung Neurochemie, Max-Planck-Institut für HirnforschungDeutschordenstrasse 46, 60528 Frankfurt, Germany

**Keywords:** glycine receptor, hereditary hyperekplexia, spa/spa TG456 mice, startle syndrome

## Abstract

Startle disease or hereditary hyperekplexia has been shown to result from mutations in the α_1_-subunit gene of the inhibitory glycine receptor (GlyR). In hyperekplexia patients, neuromotor symptoms generally become apparent at birth, improve with age, and often disappear in adulthood. Loss-of-function mutations of GlyR α or β-subunits in mice show rather severe neuromotor phenotypes. Here, we generated mutant mice with a transient neuromotor deficiency by introducing a GlyR β transgene into the spastic mouse (spa/spa), a recessive mutant carrying a transposon insertion within the GlyR β-subunit gene. In spa/spa TG456 mice, one of three strains generated with this construct, which expressed very low levels of GlyR β transgene-dependent mRNA and protein, the spastic phenotype was found to depend upon the transgene copy number. Notably, mice carrying two copies of the transgene showed an age-dependent sensitivity to tremor induction, which peaked at ∼ 3–4 weeks postnatally. This closely resembles the development of symptoms in human hyperekplexia patients, where motor coordination significantly improves after adolescence. The spa/spa TG456 line thus may serve as an animal model of human startle disease.

## Introduction

Distinct hereditary neuromotor disorders have been shown to be due to disinhibition of motoneurons resulting from mutations in the receptor for the inhibitory amino acid neurotransmitter glycine (GlyR). Amino acid substitutions in the ligand binding α_1_-subunit of the GlyR have been identified in both genetically recessive and dominant forms of hereditary hyperekplexia or startle syndrome (Shiang *et al*. 1993;Rees *et al*. 1994). This disease is characterized by startle-induced, generalized muscle contractions, which are often accompanied with hypertonia. Severe forms can be detected already at birth and are known as stiff baby syndrome (Lingam *et al*. 1981). The disease phenotype correlates well with the functional role and the anatomical localization of the GlyR, the major inhibitory ligand-gated chloride channel in the spinal cord and brain stem. In the adult spinal cord, the GlyR is composed of three copies of the ligand-binding α_1_-subunit and two copies of the structural β-subunit (Langosch *et al*. 1988;Kuhse *et al*. 1993); the latter targets the GlyR to the postsynaptic membrane by interaction with the cytoskeletal-anchoring protein gephyrin (Kirsch *et al*. 1995;Meyer *et al*. 1995). During development and in other brain regions, additional differentially expressed α-subunit isoforms (α_2_–α_4_) create GlyR diversity (Grenningloh *et al*. 1990a;Kuhse *et al*. 1990;Malosio *et al*. 1991;Betz 1992;Matzenbach *et al*. 1994).

In the mouse, several mutations of GlyR-subunit genes with complete or partial loss of GlyR function have been described. A null mutation in the α_1_-subunit in the mouse oscillator (*spd*^*ot*^) produces a strong neuromotor phenotype that causes death at ∼ 3 weeks postnatally (Buckwalter *et al*. 1994). A partial loss of function is observed in the recessive mutant spasmodic (*spd*), which carries a single base pair substitution within the GlyR α_1_ gene that decreases the agonist affinity of the GlyR (Ryan *et al*. 1994;Saul *et al*. 1994). In contrast, in the recessive mouse mutant spastic (*spa*), GlyR levels are reduced to ∼ 10–20% of the wild-type level, but the receptor is pharmacologically normal (White & Heller 1982;Becker *et al*. 1986). This phenotype is due to an insertion of a repetitive LINE 1 element in the GlyR β gene, leading to deficient mRNA splicing with functional β mRNA levels being decreased to ∼ 10% (Kingsmore *et al*. 1994;Mülhardt *et al*. 1994). Because the β-subunit is required for the synaptic localization of the receptor (Kirsch *et al*. 1995;Meyer *et al*. 1995), drastically lowered receptor levels are found in postsynaptic membrane specializations of spastic mice (White & Heller 1982;Becker *et al*. 1986;Mülhardt *et al*. 1994). Homozygous *spa*/*spa* and *spd*/*spd* mice display complex neuromotor phenotypes characterized by an exaggerated startle response, an impaired righting reflex, the development of characteristic tremors and reduced male fertility. These disease symptoms become manifest at ∼ 2 weeks of age, i.e. the time when neonatal α_2_ GlyRs are lost from the spinal cord and brain stem of normal mice (Becker *et al*. 1988,^1992^).

In an earlier study, we have reported that the *spa*/*spa* phenotype can be rescued by transgenic expression of an exogenous rat GlyR β minigene (Hartenstein *et al*. 1996). This experiment proved the causal relationship between the GlyR β gene mutation and the spastic phenotype, and showed that mRNA expression levels corresponding to ∼ 25% of that seen in wt mice suffice to completely rescue the disease symptoms. Here we describe a novel mouse strain expressing even lower levels of the rat β transgene that displays a transient phenotype, which closely resembles the symptoms characterizing the most common forms of hereditary hyperekplexia in humans (Suhren *et al*. 1966;Andermann *et al*. 1980;Kurczynski 1983). These mice might provide an animal model that may help to develop novel strategies for therapeutic intervention in hyperekplexia and spasticity.

## Materials and methods

### Generation of the transgenic line TG456

The fragment used for microinjection containing the rat GlyR-subunit cDNA under the control of the rat NSE-promotor ( Forss-Petter *et al*. 1990) and the poly(A) site of the SV40-small T antigen including its splice-site (Gorman *et al*. 1982) has been described earlier (Hartenstein *et al*. 1996). Microinjection into pronuclei of zygotes generated from F1 hybrid female mice (C57bl/6 × DBA/2) mated to DBA/2 males was performed according to published procedures (Hogan *et al*. 1986). The transgenic founder TG456 was identified by Southern blot analysis and used to breed mice of the TG456/+ genotype.

### Generation of TG456 animals on spa/spa background

Homozygous *spa*/*spa* mice were bred by intercrossing heterozygous *spa*/+ breeders (B6C3Fe-*a*/*a*-*spa*/+) purchased from Jackson Laboratory (Bar Harbor, ME, USA). For the rescue experiments, homozygous *spa*/*spa* females were crossed with TG456/+ males. Transgenic littermates of the F1 generation were then intercrossed to obtain transgenic *spa*/*spa* mice. These were intercrossed further to obtain animals of all relevant genotypes: *spa/spa*TG456/+, *spa*/*spa*TG456/TG456, and *spa*/*spa* non-transgenic littermates.

### Genotyping and mRNA analysis

The isolations of DNA from tail biopsies and mRNA from tissue as well as PCR, Southern, Northern and dot blot techniques were performed as described in Hartenstein *et al*. (1996). For genotyping, the ^32^P-labelled Dra II fragment of the rat GlyR β-subunit cDNA (Grenningloh *et al*. 1990b) was used as a hybridization probe. Northern blotting was performed with a 264-bp SspI–NcoI fragment representing exons 4 and 5 of the GlyR β cDNA. The *spa* allele was followed by allele-specific PCR using the primers described inMülhardt *et al*. (1994).

### Western blot and strychnine binding analysis

The preparation of membranes from the spinal cord and brain stem, and the solubilization, extraction and precipitation of membrane proteins were performed as described in Becker *et al*. (1986). For sodium dodecyl sulphate–polyacrylamide gel electrophoresis (SDS–PAGE), membrane pellets were suspended in Laemmli sample buffer and heated to 95 °C for 5 min.; 10–15 μg of total protein per lane was then electrophoresed on a 10% SDS–polyacrylamide gel, blotted onto Immobilon-P membrane (Millipore, Eschborn, Germany), and reacted with the monoclonal antibodies GlyR mAb4a (Becker *et al*. 1993) and anti-synaptophysin (anti-SVP, S-5768, Sigma, München, Germany). Bound immunoglobulins were visualized with horseradish peroxidase-conjugated anti-mouse IgG using the ECL system (Amersham, Braunschweig, Germany).

Binding of ^3^[H]-strychnine (NEN Du Pont, Köln, Germany) to membrane fractions from the brain was performed essentially as described (Pfeiffer & Betz 1981;Becker *et al*. 1986).

### Phenotype characterization by handling

Animals were examined over a time period of 2–4 weeks two to three times a week by several handling tests. Inducible tremor and hind feet clasping behaviour was monitored after lifting the animals by their tails. The righting response was quantitated as follows. Animals were brought into a supine position by twitching the tail; the time that elapsed after release of the tail until resuming an upright position was measured by a second investigator; and each test was repeated in three consecutive trials.

### Electromechanical tremor recording

For tremor recording, the mice were fixed by their tail to a F30 force transducer (Type 372) connected to a bridge amplifier (Type 336, both from Hugo Sachs Elektronik, 79229 March-Hugstetten, Germany) using a 5-cm thread of sewing silk. Electric signals were recorded by a conventional recorder ( Hartenstein 1996). All procedures were approved by the Regierungspräsidium Karlsruhe.

## Results

### Generation of the mouse strain spa/spa-TG456

Transgenic mice expressing the rat GlyR β gene thoughout the central nervous system were generated using an expression construct described earlier ( Hartenstein *et al*. 1996), in which the rat β cDNA was joined to the neuron-specific enolase (NSE) promoter from rat and flanked by the SV40 small T antigen polyadenylation signal. The NSE promoter has been shown to provide brain-specific expression of transgenes, however, large variations in expression efficiency have been reported (Forss-Petter *et al*. 1990;Hartenstein *et al*. 1996). Founder animals carrying this transgene were crossed into the *spa*/*spa* background. One of these founder strains, TG456, displayed a particularly interesting phenotype; in contrast to other transgenic strains carrying the same construct (Hartenstein *et al*. 1996), a haploid copy of the TG456 transgene did not rescue the phenotype. However, when spa/^+^-TG456/^+^ animals were intercrossed, several animals homozygous for the *spa* allele showed intermediate phenotypes displaying noticeably alleviated symptoms. This suggested that their phenotype might depend on gene dosage and prompted us to study the correlation between transgene copy number and phenotype in such mice in more detail.

### Transgene expression in spa/spa-TG 456 mice

Animals resulting from double heterozygous crosses (see above) were first analysed for their transgene status by dot blot hybridization on genomic DNA with a rat GlyR β cDNA fragment (data not shown). Adult animals displaying a transgene hybridization signal twofold higher than those obtained with TG456/+ mice consistently showed a less severe spastic phenotype than non-transgenic *spa*/*spa* littermates (see below for detailed analysis). Thus, a gene dosage effect on the *spa* phenotype expression was clearly present. To monitor the corresponding transgene expression levels, we performed Northern analyses on brain mRNA of *spa*/*spa*-TG 456/^+^ and *spa*/*spa*-TG456/TG456 transgenic mice, respectively. Because the GlyR β RNA transcribed from the endogenous *spa* allele is < 10% functional and > 90% aberrantly spliced (Mülhardt *et al*. 1994), we used a probe covering exons 4 and 5 which does not detect the aberrantly spliced endogenous GlyR β RNA (Hartenstein *et al*. 1996). The transgene-specific band should comigrate with the endogenous 3.5-kb mature functional mRNA. Five independent Northern analyses on the four genotypes wt, *spa*/*spa*, *spa*/*spa*-TG456/+, and *spa*/*spa*-TG456/TG456 were performed, one of which is depicted inFig. 1(A). When the hybridization signal intensities were analysed, of all of them only very subtle differences between *spa*/*spa and* the transgenic lines were detected. We therefore concluded that the transgene-specific GlyR β mRNA expression was very low.

**FIG. 1:**
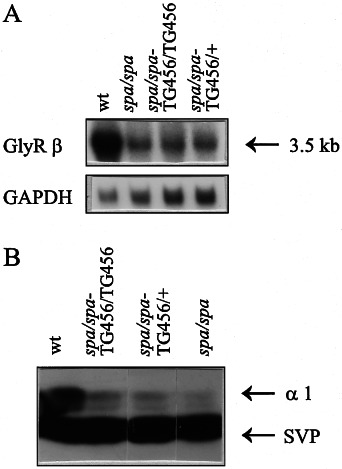
Expression of the transgene in *spa*/*spa* mice hetero- and homozygous for the TG 456 insertion. (A) Northern analysis. Total brain RNA was probed with a 264-bp SspI–NcoI fragment encompassing exons 4 and 5 of the GlyR β cDNA. Control hybridizations of the same blot were performed using the GAPDH gene probe. wt, wild-type control mice. (B) Western analysis. Membrane proteins from spinal cord and brain stem were separated on SDS–PAGE gels, transferred to nitrocellulose, and specific bands were detected with the antibodies mAb 4a and anti-SVP against synaptophysin as a control. Immunoglobulins were visualized using horseradish peroxidase-coupled second antibodies and the ECL system (Amersham).

**FIG. 2:**
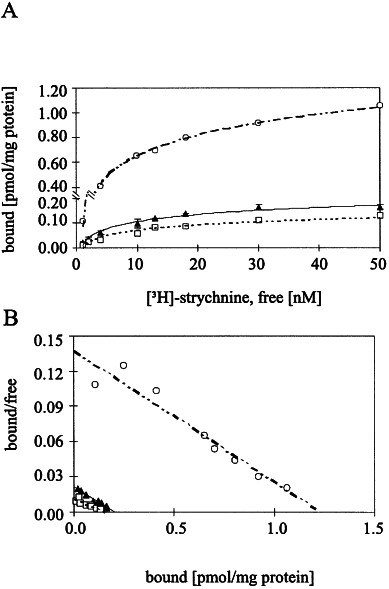
Binding of ^3^[H] strychnine to spinal cord membranes. Genotypes: (○), wt; (□), *spa*/*spa;* and (▴), *spa*/*spa*-TG456/TG456. Data represent the means from two (wt, *spa*/*spa*) or three (*spa*/*spa*-TG456/TG456) animals, respectively. (A) Saturation binding curves and (B) Scatchard analyses of specific ^3^[H]-strychnine binding assays were performed in triplicate as described inBecker *et al*. (1993).

In order to investigate the GlyR protein levels in the transgenics, we performed Western blot analyses and ligand-binding assays. While suitable anti β-subunit antibodies did not exist, in Western blots the antibody mAb 4a was used, which recognizes mainly the 48-kDa α_1_-subunit within the adult receptor (Pfeiffer *et al*. 1984;Kirsch *et al*. 1995). Under conditions of limiting β-subunit expression, as in adult *spa*/*spa* mice, membrane-bound mAb 4a immune reactivity is a valid measure of β-subunit surface expression and GlyR complex formation, because stable expression of the adult α_1_-subunit on the neuronal surface requires β-subunit expression in animals (Becker *et al*. 1986; 1992;Hartenstein *et al*. 1996).Figure 1(B) shows that consistent with the mRNA analysis, *spa*/*spa*-TG456/+ as well as *spa*/*spa*-TG456/TG456 animals express membrane-bound 48 α_1_-subunit at about the same levels as non-transgenic *spa*/*spa* mice. To enhance the sensitivity of the detection and at the same time assess the GlyR function we performed binding assays with the competitive GlyR antagonist strychnine.Figure 2(A) shows the isotherm for binding of ^3^[H]-strychnine to membrane preparations of wt, *spa*/*spa* homozygotes and *spa*/*spa*-TG 456/TG456 mice. Compared with wt mice, the *spa*/*spa* and *spa*/*spa*-TG456/TG456 animals both showed very low levels of binding. However, in triplicate measurements the latter genotype appeared to show consistently higher binding than *spa*/*spa* animals. Scatchard analysis of these data (Fig. 2B) indicated that in all three genotypes tested the affinity of the GlyR is very similar. The *K*_D_ values for wt, *spa*/*spa*-TG456/456, and non-transgenic *spa*/*spa* animals were 10.4, 9.5 and 10.5 nm, respectively. The corresponding *B*_max_ values were 1271 (wt), 173 (*spa*/*spa*-TG456/456) and 166 (*spa*/*spa*) fmoles/mg protein. In conclusion, the expression of the transgene in TG456 mice is very low.

### Correlation of transgene dosage with motor phenotype

To unravel whether the phenotypic variations observed in the transgenic animals could be correlated to transgene dosage, we quantified the phenotypic properties of *spa*/*spa* animals hetero- and homozygous for the TG456 transgene more systematically. To this end, we performed a few simple handling assays. Firstly, we screened animals of different genotypes daily for the development of tremors. As depicted inFig. 3, in non-transgenic *spa*/*spa* mice the onset of tremor inducibility started at ∼ 14–17 days old and lasted throughout adulthood. This time of onset of handling-induced tremor proneness coincides with the replacement of α_2_-subunit-containing neonatal by α_1_-subunit-containing adult GlyRs (Becker *et al*. 1988; 1992). In *spa*/*spa*-TG456/+ mice carrying one copy of the transgene 456, tremor inducibility was not significantly altered. However, animals homozygous for the transgene became tremor prone only after 20–35 days. Notably, tremor inducibility was only transient; strong tremor was seen only over a few (2–10) days. Thereafter, the *spa*/*spa*-TG456/TG456 animals displayed tremor development upon handling only rarely.

**FIG. 3:**
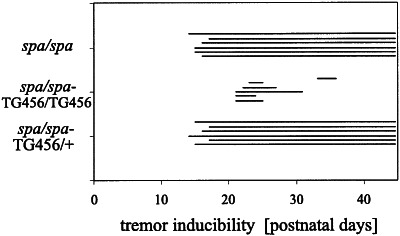
Tremor inducibility in *spa*/*spa*, *spa*/*spa*-TG456 and *spa*/*spa*-TG456/TG456 animals. Animals of each genotype were tested at several time points for tremor inducibility by handling. Periods of tremor inducibility are indicated by solid lines. Note delayed and transient appearance of tremor symptoms in *spa*/*spa*-TG456/TG456 mice. Of the non-transgenic *spa*/*spa* animals, four were non-transgenic littermates from doubly heterozygous crosses, and three animals were derived from the original stock obtained from Jackson Laboratories.

**FIG. 4:**
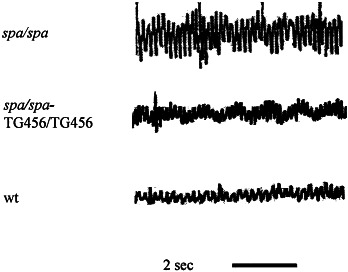
Electromechanical tracings of tremor-derived movement recorded from *spa*/*spa*, *spa*/*spa*-TG456/TG456 and wt animals. The amplitude is proportional to the strength of the movement recorded over time. For experimental details see text and Materials and methods.

Tremor induction can be easily sensed when spastic animals are picked up by the tail. To monitor the tremor we used an electromechanical transducer onto which the mouse was fixed by the tail. Vibration resulting from tremor could then be recorded over time as apparent weight changes, with the amplitudes of the tracings reflecting the intensity of the movements. Figure 4 shows that two copies of the TG456 transgene strongly reduced the tremor-specific amplitudes recorded from *spa*/*spa* mice.

As a second parameter to monitor motor performance, we measured the mean righting time by turning the animals on their back and determining the time required to regain an upright position. With this assay, gradual differences in the righting response can be observed ( Hartenstein *et al*. 1996). Here, we only quantified whether the animals regained their normal position in < 1 s. The presence of only a single copy of the transgene markedly influenced this parameter (Tables 1, 58% of measurements at different times on seven animals). In mice homozygous for the transgene, rescue was observed in the vast majority of these animals (86% of measurements on 28 individuals). In contrast, in non-transgenic *spa*/*spa* animals we never observed righting in < 5 s; most *spa*/*spa* mice required > 30 s to perform this task (see alsoHartenstein *et al*. 1996).

**Table 1:** Phenotype characteristics of the different mouse lines

	*spa/spa*	*spa*/*spa*-TG456/+	*spa*/*spa-*TG456/TG456	wt
Animals tested (*n*)	10	7	28	10
Hind feet clasping (%)	100	71	32	0
Righting time ≤ 1 s (%)	0	58	86	100

Of the non-transgenic *spa*/*spa* animals, four were non-transgenic littermates from doubly heterozygous crosses, and six animals were derived from the original stock obtained from Jackson Laboratories.

A third criterion to evaluate neuromotor performance was the so-called ‘hind feet clasping’ phenomenon which represents an abnormal behaviour of *spa*/*spa* homozygotes which clasp their hind feet when picked up by the tail (Buckwalter *et al*. 1994). This phenotype was rescued in two-thirds of the *spa*/*spa*-TG456/TG456 double homozygotes and in one-third of the *spa*/*spa*-TG456/+ heterozygous transgenics (Table 1). Notably, hind feet clasping was still observed in several of the *spa*/*spa*-TG456/TG456 animals that no longer showed induced tremor (seeFig. 3). Thus, the hind feet clasping behaviour appears to be a more sensitive parameter to monitor reduced GlyR levels than tremor development.

Finally, we observed profound differences in male fertility correlating with transgene dosage. *Spa*/*spa* males are known to perform very poorly in siring offspring. Although we did not precisely quantify reproductive performance, we also experienced this in our mouse colony. By contrast, *spa*/*spa*-TG456/TG456 males displayed fertility indistinguishable from wt studs, while heterozygous transgene carriers showed an intermediate phenotype in this respect.

The behavioural and motor performance of mutant phenotypes may not only depend on the expression of the gene in question but also on the genetic background ( Logue *et al*. 1997;Kelly *et al*. 1998). Because the breeding of the transgene into the *spa*/*spa* genetic background produced genetic hybrid strains, we had to establish whether this by itself could possibly alleviate the *spa*/*spa* phenotype. Therefore, we included both the original *spa*/*spa* strain from Jackson Laboratories and non-transgenic *spa*/*spa* littermates from our transgene breedings into our experiments. In particular, for the analyses summarized inTable 1 four out of 10 *spa*/*spa* mice were non-transgenic littermates. Into the tremor analysis depicted inFig. 3, also four non-transgenic littermates were included. We found that with respect to the behavioural assays performed here, non-transgenic *spa*/*spa* mice on both genetic backgrounds behaved indistinguishably, thus allowing us to treat them as one experimental group (seeFig. 3 andTable 1). These data corroborate our earlier results derived from analogous breedings of two other GlyR β-transgenic *spa*/*spa* lines, in which we never observed a phenotypic modification in non-transgenic littermates (Hartenstein *et al*. 1996;Hartenstein 1996). In summary, we therefore conclude that the behavioural modifications observed here were dependent upon the presence and copy number of the low-expression transgene TG456. In addition, the different disease symptoms were differentially affected by the transgene dosage.

## Discussion

In this paper, we describe a mouse strain transgenic for the rat GlyR β-subunit gene expressed at very low levels. This transgene can modify by partial rescue the phenotype of the spastic mutation in a gene dosage-dependent manner. It revealed that the rescue of the different behavioural and motor deficits showed distinct transgene dosage requirements. For example, in *spa*/*spa*-TG456/+ heterozygotes, no rescue was seen with respect to tremor inducibility, whereas male fertility, hind feet clasping and righting response were clearly improved. Furthermore, in TG456 homozygotes male fertility and righting response were completely rescued, whereas tremor induction and hind feet clasping behaviour were only partially improved. Our data also show that the different deficiencies appeared independently of each other, e.g. hind feet clasping did not require a defective righting response or tremor proneness. Thus, the different symptoms may originate from deficiencies at distinct sites of GlyR action.

The age dependence of tremor inducibility in *spa*/*spa* mice correlates with the appearance of the adult and the disappearance of the neonatal GlyR during the postnatal life of rodents (Becker *et al*. 1988). In *spa*/*spa*-TG456/TG456 mice, the onset of tremor inducibility was not only delayed but also of a transient nature. This is particularly interesting because alleviation of hyperexcitability with age is also seen in human hereditary hyperekplexia. A remarkable parallel thus appears to exist between mouse and man in the developmental dependence on efficient GlyR function.

The relatively subtle phenotype of *spa*/*spa*-TG456/TG456 animals constitutes a much better animal model of human GlyR disease than that seen in the spontaneous spastic mutant. Because of the high sensitivity of the disease phenotype to changes in GlyR levels, these mice might be of value for pharmacological studies. For example, although clonazepam and valproate have been successfully implemented in the therapy of human hyperekplexia (Dooley & Andermann 1989;Ryan *et al*. 1992), the exact mechanisms of action of these drugs on the organism level are still not clear.

In startle disease patients, characteristic symptoms, e.g. touch- or sound-induced tremor formation are often detectable already at birth, resembling the so-called ‘stiff baby syndrome’. In spastic and spasmodic mutant mice, however, motor deficiencies appear only between 2 and 3 weeks of postnatal life, i.e. a period corresponding to adolescence. This relative delay in mice may be attributed to the postnatal expression of the neonatal α_2_-subunit, which can form functional homo-oligomeric GlyRs (Hoch *et al*. 1989;Schmieden *et al*. 1992). In humans, a homologue of the α_2_-subunit exists (Grenningloh *et al*. 1990a), but nothing is known about its temporal expression pattern in early childhood.

Another difference between the human GlyR disease and the phenotype of *spa*/*spa* mice concerns the strongly reduced fertility of male mice. Consistent with the motor deficiencies seen, this could reflect an inability of *spa*/*spa* males to mount females. An alternative explanation is offered by the recent identification of GlyR expression in porcine sperm (Melendrez & Meizel 1996). Also, strychnine has been shown to block *in vitro* the acrosome reaction of porcine and human sperm (Melendrez & Meizel 1995). Whatever the underlying mechanisms might be, in *spa*/*spa*-TG456/TG456 animals the male fertility is normal. Again, this particular genotype resembles human startle disease in that no fertility problems have been reported for such patients.

There are dominant and recessive forms of hereditary hyper-ekplexia in man. At the molecular level, the *spa*/*spa*-TG456 mice represent a partial loss of function model and therefore may be regarded as correlates of the recessively inherited form of the human disease. The more frequent forms of hyperekplexia are dominantly inherited. The respective mutant GlyR α_1_ subunits display dominant negative effects upon heterologous expression in mammalian cell lines and Xenopus oocytes (Rajendra *et al*. 1994;Langosch *et al*. 1994). The consequences of expression of such mutations may be explored further by introducing such human hyperekplexia antimorphs as transgenes into mice.

## Abbreviations

GlyR: glycine receptor
NSE: neuron-specific enolase
SDS–PAGE: sodium dodecyl sulphate–polyacrylamide gel electrophoresis
